# Novel 1,3,4-Oxadiazole Derivatives of Pyrrolo[3,4-*d*]pyridazinone Exert Antinociceptive Activity in the Tail-Flick and Formalin Test in Rodents and Reveal Reduced Gastrotoxicity

**DOI:** 10.3390/ijms21249685

**Published:** 2020-12-18

**Authors:** Marta Szandruk-Bender, Benita Wiatrak, Łukasz Szczukowski, Piotr Świątek, Maria Rutkowska, Stanisław Dzimira, Anna Merwid-Ląd, Maciej Danielewski, Adam Szeląg

**Affiliations:** 1 Department of Pharmacology, Faculty of Medicine, Wroclaw Medical University, Mikulicza-Radeckiego 2, 50-345 Wrocław, Poland; benita.wiatrak@umed.wroc.pl (B.W.); maria.rutkowska@umed.wroc.pl (M.R.); anna.merwid-lad@umed.wroc.pl (A.M.-L.); maciej.danielewski@umed.wroc.pl (M.D.); adam.szelag@umed.wroc.pl (A.S.); 2 Department of Chemistry of Drugs, Faculty of Pharmacy, Wroclaw Medical University, Borowska 211, 50-556 Wrocław, Poland; lukasz.szczukowski@umed.wroc.pl (Ł.S.); piotr.swiatek@umed.wroc.pl (P.Ś.); 3 Department of Pathology, Faculty of Veterinary Medicine, Wroclaw University of Environmental and Life Sciences, Norwida 31, 50-375 Wrocław, Poland; stanislaw.dzimira@upwr.edu.pl

**Keywords:** pain, tail-flick test, formalin test, pyrrolo[3,4-*d*]pyridazinone, 1,3,4-oxadiazole

## Abstract

Despite the availability of the current drug arsenal for pain management, there is still a clinical need to identify new, more effective, and safer analgesics. Based on our earlier study, newly synthesized 1,3,4-oxadiazole derivatives of pyrrolo[3,4-*d*]pyridazinone, especially **10b** and **13b**, seem to be promising as potential analgesics. The current study was designed to investigate whether novel derivatives attenuate nociceptive response in animals subjected to thermal or chemical noxious stimulus, and to compare this effect to reference drugs. The antinociceptive effect of novel compounds was studied using the tail-flick and formalin test. Pretreatment with novel compounds at all studied doses increased the latency time in the tail-flick test and decreased the licking time during the early phase of the formalin test. New derivatives given at the medium and high doses also reduced the late phase of the formalin test. The achieved results indicate that new derivatives dose-dependently attenuate nociceptive response in both models of pain and exert a lack of gastrotoxicity. Both studied compounds act more efficiently than indomethacin, but not morphine. Compound **13b** at the high dose exerts the greatest antinociceptive effect. It may be due to the reduction of nociceptor sensitization via prostaglandin E_2_ and myeloperoxidase levels decrease.

## 1. Introduction

The primary function of pain is its warning and protective role. It forewarns about threatening tissue damage due to injury or disease and triggers the organism’s reflex and behavioral response to minimize the effects of this damage [1]. The importance of such behaviors is well illustrated in the pathological cases of congenital insensitivity to painful stimuli, in which natural experiences can have disastrous consequences [2]. If tissue damage is unavoidable, then, both in the peripheral and central nervous systems, changes occur expressing in pain and hypersensitivity as a response to inflammation in the tissues surrounding nerve structures. Appropriate analgesic management, as well as natural tissue healing processes, cause that acute pain to disappear after a few or several days. However, in the absence or ineffective analgesic treatment, persistent pain leads to pathophysiological changes in the nervous system, and the acute form of pain can develop into chronic pain syndrome that loses its warning nature and becomes not even a symptom, but a disease in itself [1,2].

Patients suffering from pain often receive inadequate pain relief from current medications. Since the current pharmacotherapy, including non-steroidal anti-inflammatory drugs (NSAIDs) and opioids, has substantial limitations as regards to safety and efficacy, there is still an unmet clinical need for the identification of new, more efficient, and safer molecules to pain management [3].

One of the approaches in medicinal chemistry is to combine structure, characterized by known analgesic properties, with a pharmacophore capable of enhancing this activity. The pyrrolo[3,4-*d*]pyridazinone core is a structure that exerts analgesic activity [4,5]. In turn, diversely substituted five-membered rings of 1,3,4-oxadiazole-2-thione, which is a bioisosteric analogue of the carboxylic group, presents various biological activities, including antinociceptive and anti-inflammatory [6,7,8,9]. Worldwide used analgesics, e.g., ibuprofen or diclofenac, were modified in this way. The obtained derivatives of the mentioned drugs, possessing in their structure 1,3,4-oxadiazole-2-thione ring, show significant analgesic and anti-inflammatory activity and diminished adverse gastrointestinal effects.

**Figure 1:**
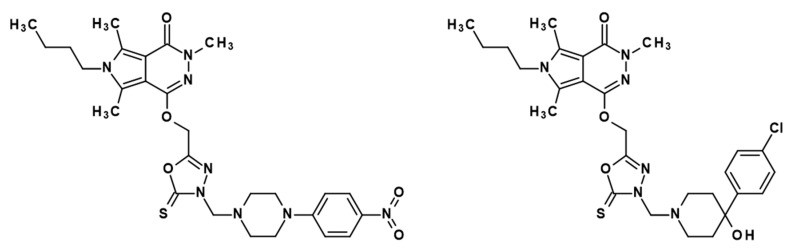
Chemical structures of investigated compounds **10b** (**left**) and **13b** (**right**).

In our previous study, we have reported the design, synthesis, and comprehensive biological evaluation of new 1,3,4-oxadiazole derivatives of pyrrolo[3,4-*d*]pyridazinone [6]. According to performed in vitro and in silico investigations, new molecules exert good cyclooxygenase (COX) inhibition activity with superior affinity to COX-2 isoenzyme [6,10]. Encouraged by achieved results, we decided to assess the most promising compounds, **10b** and **13b**, in in vivo models of nociception. Structures of studied derivatives are presented in Figure 1.

The current study was undertaken to elucidate the impact of two newly synthesized pyrrolo[3,4-*d*]pyridazinone derivatives, **10b** and **13b**, on antinociceptive behavior in noxious stimuli induced models of pain, i.e., the tail-flick test in rats and formalin test in mice, and compare this effect to the reference drugs such as indomethacin and morphine. Moreover, the objective of this study was to examine the influence of new derivatives on the gastric mucosa.

## 2. Results

### 2.1. Tail-Flick Test

**Figure 2:**
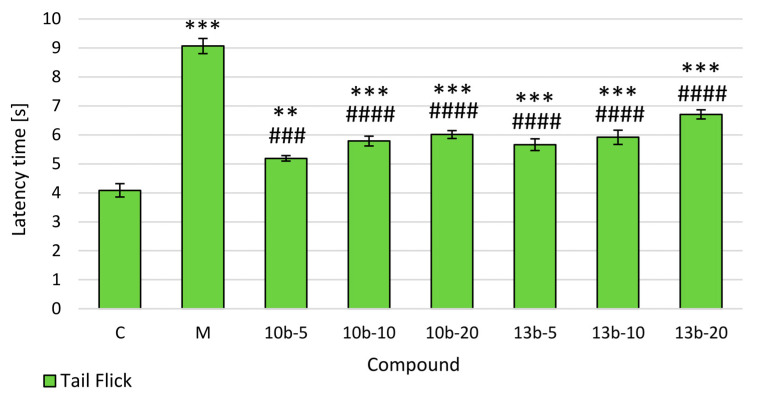
The effects of compounds **10b** and **13b** on the latency time in the tail-flick test. Morphine was used as a reference drug. All compounds were administered intragastrically. Experimental groups (*n* = 12): C—control group; M—group receiving 10 mg/kg of morphine; **10b**-5, **10b**-10, **10b**-20—groups receiving, respectively, 5, 10, or 20 mg/kg of investigated compound **10b**; **13b**-5, **13b**-10, **13b**-20—groups receiving, respectively, 5, 10, or 20 mg/kg of investigated compound **13b**. Data are presented as mean ± SEM. Differences ** *p* < 0.01 vs. control group; *** *p* < 0.001 vs. control group; ### *p* < 0.001 vs. morphine group; #### *p* < 0.0001 vs. morphine group were deemed statistically significant.

To assess the central antinociceptive activity of intragastrically administered novel compounds **10b** and **13b**, the tail-flick test was conducted, the results of which are presented in Figure 2. The pretreatment of rats with both studied compounds (5, 10, 20 mg/kg) as well as morphine showed statistically significant and dose-dependent antinociceptive activity in the tail-flick test as compared to the control group. Pretreatment with novel compounds were significantly weaker in increasing the latency time to the thermal stimulation compared to morphine.

### 2.2. Formalin Test

**Figure 3:**
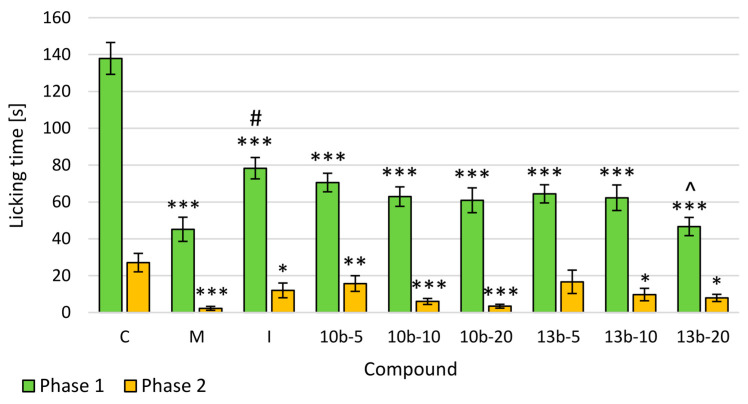
The effects of compounds **10b** and **13b** on the licking time in the early (0–5 min) and late (25–30 min) phases of the formalin test. Morphine and indomethacin were used as reference drugs. All compounds were administered intragastrically. Experimental groups (*n* = 12): C—control group; M—group receiving 10 mg/kg of morphine; I—group receiving 10 mg/kg of indomethacin; **10b**-5, **10b**-10, **10b**-20—groups receiving, respectively, 5, 10, or 20 mg/kg of investigated compound **10b**; **13b**-5, **13b**-10, **13b**-20—groups receiving, respectively, 5, 10, or 20 mg/kg of investigated compound **13b**. Data are presented as mean ± SEM. Differences * *p* < 0.05 vs. control group; ** *p* <0.01 vs. control group; *** *p* < 0.001 vs. control group; # *p* < 0.05 vs. morphine group; ^ *p* < 0.05 vs. indomethacin group were deemed statistically significant.

To assess the central and peripheral antinociceptive activity of intragastrically administered tested compounds **10b** and **13b**, the formalin test was performed, the results of which are presented in Figure 3. The pretreatment of mice with tested or reference compounds resulted in an inhibition of formalin-induced licking both in the early and late phases of the formalin test. The tested compounds and reference drugs (morphine and indomethacin) had a statistically significant effect on the time the mice licked the paw (the pain passed fast) in the early phase. In the early phase, the maximum antinociceptive effect compared to the control group was observed after administration of morphine at the dose of 10 mg/kg (statistically significant) and compound **13b** at the dose of 20 mg/kg, which was comparable to the effect of morphine. Moreover, the antinociceptive activity of compound **13b** at the dose of 20 mg/kg was statistically significantly greater than that of indomethacin, which was statistically significantly weaker compared to morphine. In the late phase, the highest and statistically significant antinociceptive effect (compared to the control group) was demonstrated by both novel compounds at the doses of 10 and 20 mg/kg and morphine. The dose-dependence of the tested compounds, **10b** and **13b**, was demonstrated in the formalin test, both in the early and late phases.

### 2.3. Evaluation of Prostaglandin E_2_ (PGE_2_) and Myeloperoxidase (MPO) Levels

**Figure 4:**
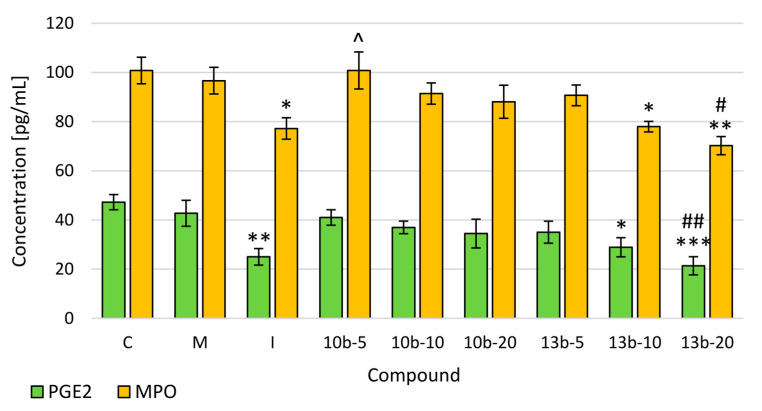
The effects of compounds **10b** and **13b** on the levels of Prostaglandin E2 (PGE_2)_ and Myeloperoxidase (MPO) in mice serum. Blood samples were collected before mice were sacrificed. Morphine and indomethacin were used as reference drugs. Experimental groups (*n* = 12): C—control group; M—group receiving 10 mg/kg of morphine; I—group receiving 10 mg/kg of indomethacin; **10b**-5, **10b**-10, **10b**-20—groups receiving, respectively, 5, 10, or 20 mg/kg of investigated compound **10b**; **13b**-5, **13b**-10, **13b**-20—groups receiving, respectively, 5, 10, or 20 mg/kg of investigated compound **13b**. Data are presented as mean ± SEM. Differences * *p* < 0.05 vs. control group; ** *p* < 0.01 vs. control group; *** *p* <0.001 vs. control group; # *p* < 0.05 vs. morphine group; ## *p* < 0.01 vs. morphine group; ^ *p* < 0.05 vs. indomethacin group were deemed statistically significant.

The enzyme-linked immunosorbent assay (ELISA) tests were performed to evaluate the levels of PGE_2_ and MPO, the increase of which takes place in the nociception caused by the inflammatory process. The level of PGE_2_ was the lowest after compound **13b** administration at the dose of 20 mg/kg and indomethacin. In the MPO level, a statistically significant decrease was demonstrated after the application of compound **13b** at the doses of 10 and 20 mg/kg and indomethacin. At the same time, after compound **13b** administration at the dose of 20 mg/kg, statistically, significantly lower levels of PGE_2_ and MPO were observed compared to morphine. In the case of both compounds, **10b** and **13b**, a dose-dependence was demonstrated for PGE_2_ and MPO levels (Figure 4).

### 2.4. Histopathological Assessment of Gastric Mucosa

**Figure 5:**
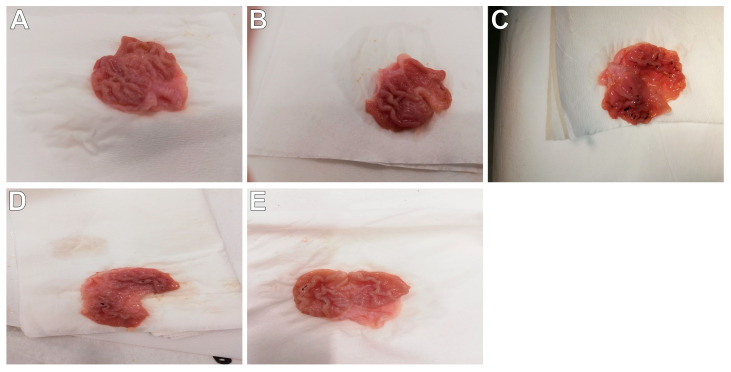
Macroscopic appearance of the gastric mucosa demonstrated that studied compounds caused negligible mucosal lesions. Morphine and indomethacin were used as reference drugs. Experimental groups (*n* = 12): Control group (**A**); group receiving 10 mg/kg morphine (**B**); group receiving 10 mg/kg indomethacin (**C**); group receiving 20 mg/kg compound **10b** (**D**); group receiving 20 mg/kg compound **13b** (**E**).

**Figure 6:**
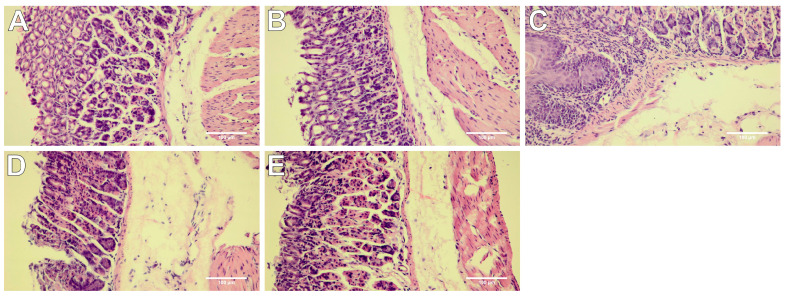
Microscope appearance of stomach tissue after hematoxylin-eosin (H&E) staining demonstrated that studied compounds neither altered the normal stomach architecture nor induced ulcerations. Morphine and indomethacin were used as reference drugs. Experimental groups (*n* = 12): Control group (**A**); group receiving 10 mg/kg morphine (**B**); group receiving 10 mg/kg indomethacin (**C**); group receiving 20 mg/kg compound **10b** (**D**); group receiving 20 mg/kg compound **13b** (**E**); magnification 200×.

To characterize the gastric safety profile of the studied compounds, macro- and microscopic assessments were performed. The presence and severity of macroscopically visible lesions (petechiae, hemorrhagic erosions) were scored as indicators of ulcerogenic activity. The results of these ulcerogenic liability studies demonstrated that novel derivatives in all studied dose ranges caused negligible stomach lesions (*p* = NS, Table 1, Figure 5D,E), whereas indomethacin at the dose of 10 mg/kg caused significant stomach injuries (*p* < 0.001; Table 1, Figure 5C). Microscopic assessment reflected the values obtained macroscopically. The stomach tissue of mice pretreated with novel compounds as well as control mice showed no histopathological changes (Table 1, Figure 6A,D,E). Meanwhile, the stomach tissue of indomethacin-treated mice was characterized by appreciable damage of the protective mucosal layer with focal necrosis of gastric mucosa, submucosal edema, and congestion of mucosal and submucosal blood vessels (Table 1, Figure 6C). Morphine at the dose of 10 mg/kg induced no significant injury in the stomach tissue both in the macro-and microscopic analysis (Table 1, Figure 5B and Figure 6B).

### 2.5. Multi-Criteria Decision Analysis (MCDA)

**Figure 7:**
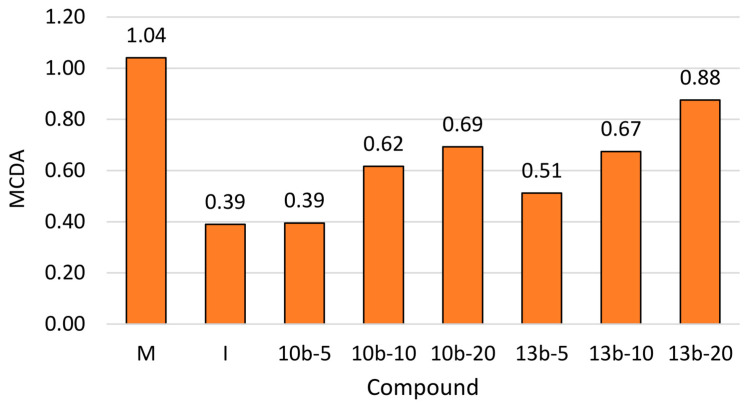
Multi-criteria decision analysis (MCDA) of the antinociceptive effect of studied compounds **10b** and **13b**.

The results obtained in the tail-flick test, formalin tests, and ELISA performed for two newly synthesized pyridazinone derivatives, as well as morphine and indomethacin, were analyzed by MCDA to compare the antinociceptive activity of novel compounds. The MCDA results (Figure 7) showed that both tested compounds acted dose-dependently. Compounds **10b** and **13b** exerted a greater antinociceptive effect than indomethacin when given at the same dose as indomethacin (10 mg/kg) and at the higher dose than indomethacin (20 mg/kg). Compound **10b** at the dose lower than indomethacin (5 mg/kg) exerted the same antinociceptive effect as indomethacin and compound **13b**—even more pronounced than indomethacin. In the studied doses range, compound **13b** exerted a greater antinociceptive effect than compound **10b**. Further, the strongest overall effect was found for morphine at the dose of 10 mg/kg, followed by compound **13b** at the dose of 20 mg/kg.

## 3. Discussion

Nociception, the term coined by Sherrington, is the neural process of detecting noxious stimuli followed by a reflex withdrawal, while pain is defined as a subjective, unpleasant, and multidimensional experience associated with actual or impending tissue damage [11,12]. Nociceptive reflexes and pain perception are tightly linked processes. Nociceptors respond to different forms of noxious stimuli (i.e., thermal, chemical, mechanical) and elicit withdrawal reflexes, which became the substitute for human pain experiences in animal models [3,12]. Even though the experimental models of nociception have limitations and translation of basic science data into effective analgesics is far from perfect, such models constitute valuable pharmacological tools used in preclinical studies to assess the potential analgesic activity of newly synthesized compounds [3,13]. In the current study, the measurement of the nociceptive response to thermal and chemical noxious stimuli have been used to elucidate novel substances, **10b** and **13b**, as potential analgesics.

One of the most common technique to assess antinociceptive activity of any novel substance is the tail-flick test, in which the measured parameter is the latency time [3,12]. Depending on the intensity of the beaming heat stimulation, the tail-flick test involves spinal and supraspinal structures. When the tail-flick apparatus leads to elicit latency reaction within 4-5 s in the intact rats (such in the current study), the tail-flick reflex becomes not only spinal, but a more complicated reaction involving higher neural structures, viz. spinobulbospinal loop [12,14]. It is noteworthy that compared to other models of thermal stimulation, the tail-flick test does not elicit tactile stimulus because beaming heat constitutes a relatively selective stimulus for nociceptors [12]. The extension of the latency time in the tail-flick test is related to the central analgesic effect of administered drugs [3,12,14]. In our study, both compounds at all tested doses (5, 10, 20 mg/kg) showed an antinociceptive effect, significantly prolonging the latency time compared to the control group in a dose-dependent manner. Their effect was weaker than that of morphine, used as a reference drug. Thus, the results obtained in the current study suggest that compounds **10b** and **13b** may modulate central nociceptive pathways.

Another model used to verify the antinociceptive effect of novel compounds is the formalin test with licking time as a measured parameter [3,12]. The formalin test comprises neurogenic, inflammatory, and central mechanisms of nociception what makes it a particularly valuable model. Moreover, in this test, the spontaneous nociceptive response in freely moving unrestrained animals with relatively long duration closely mimics clinical pain in comparison to other experimental models of nociception [13]. Subcutaneous injection of diluted formalin to the hind paw elicits a biphasic nociceptive response. The neurogenic nociception (early phase) is caused by direct activation of nociceptive afferent fibers, primarily C fibers, releasing neuropeptides such as substance P. The inflammatory nociception (late phase) is due to the inflammatory reaction caused by tissue injury with subsequent release of inflammatory mediators including prostaglandins (PGs) as well as central sensitization in the dorsal horn of the spinal cord [3,15,16]. It is assumed that functional changes in the dorsal horn are initiated by prolonged afferent input to the spinal cord following the formalin injection [17,18]. As a biphasic nociceptive response, the formalin test can be used to establish the ability of novel compounds to affect the noninflammatory (in the early phase) and inflammatory (in the late phase) associated nociceptive response. The results found in this study indicate that pretreatment with novel compounds, **10b** and **13b**, dose-dependently decreased nociceptive response relative to the control group in the early phase of the formalin test. Neither these new compounds nor indomethacin were as effective as morphine. As we earlier indicated, novel pyrrolo[3,4-*d*]pyridazinone derivatives inhibit cyclooxygenase (COX) activity [6]. It is known that in the neurogenic phase of the formalin test, substances inhibiting COX activity remain less effective in diminishing the nociceptive behavior compared to opioids [19]. Nevertheless, it should be pointed out that compound **13b** at the high dose exerted a greater antinociceptive effect than indomethacin during the early phase of the formalin test. It may suggest that in addition to the cyclooxygenase pathway, other mechanisms are also involved in the antinociceptive activity of compound **13b**. Further studies are needed to confirm this suggestion. Newly synthesized compounds also reduced the late phase of the formalin test, but only after administration at the medium and high doses. In sum, the results achieved in the formalin test suggest that compounds **10b** and **13b** are able to attenuate both neurogenic and inflammatory phases of nociceptive response. Moreover, compounds **10b** and **13b** at the medium and high doses may possess central and peripheral antinociceptive effects, while only central at the low dose. This corroborates the findings of the tail-flick test, in which centrally mediated antinociceptive activity was observed after **10b** or **13b** administration even at the low dose.

A great variety of deleterious exogenous and endogenous pathological factors such as injury, infection, autoimmune response, or tissue malfunction can trigger plenty of diverse biochemical pathways, which results in pain induction and development of an inflammatory response. The inflammatory response is, in turn, an important factor for pain perception. Inflammatory cell activation and infiltration with subsequent proinflammatory mediators release contribute to threshold decrease and strengthen the response of nociceptors that innervate damaged tissue. Hence, the hypersensitivity underlies inflammatory nociception [20,21]. Considering this expanded and complicated network of proinflammatory mediators, PGs are probably one of the best known and studied [21]. As aforementioned, formalin-induced inflammatory nociception is associated with increased prostaglandins synthesis and release [3,15,16,22]. Prostaglandins, especially PGE_2_, constitute potent sensitizing agents able to modulate the nociceptive pathway via peripheral and central mechanisms [20]. PGE_2_ is regarded as an essential mediator of hypersensitivity, which enhances the nociceptive properties of various inflammatory mediators [20,23]. Primarily, PGE_2_ increases the cAMP level and may enhance nociceptor sensitization by lowering the activation threshold of tetrodotoxin-resistant (TTX-R) sodium channels via the protein kinase A pathway [24]. Thus, prevention of PGE_2_ production and release by COX inhibitors should contribute to minimizing nociceptive response. In our previous work, we demonstrated that new pyrrolo[3,4-*d*]pyridazinone derivatives strongly inhibit cyclooxygenase with a better affinity towards COX-2 isoform [6]. Based on these findings, we expected both compounds examined in this study to lower PGE_2_ levels. Our results turned out to be a bit surprising. Compound **13b** but not **10b** at the medium and high doses decreased the level of PGE_2_ as compared to the control group. It may be explained by the fact that although both cyclooxygenase isoforms are involved in the inflammatory reaction and play an important role in the peripheral and central sensitization, COX-2 is the prevalent isoform involved in the sensitization derived from peripheral inflammation at the local, spinal, and supraspinal level [20,22,24].

The influx of neutrophils, as well as their product, myeloperoxidase, seem to participate in inflammatory nociception [25,26,27,28]. Neutrophils may lead to primary nociceptive neuron sensitization via mediating PGE_2_ release [26,27]. Inflammation entails neutrophil’s recruitment into the tissue, their activation, and thereby myeloperoxidase release. Since MPO, a member of the heme peroxidase enzyme superfamily, catalyzes reactive oxygen species (ROS) generation, neutrophils could also be involved in such mechanisms of nociception as reactive oxygen and nitrogen species (RNS) production [29,30]. The ROS and RNS are capable of acting as noxious stimuli mediators, which increase the excitability of the dorsal horn neurons. It has been demonstrated that ROS and RNS are able to decrease glutamate transporters activity and increase vanilloid and *N*-methyl-D-aspartate receptors activity [31]. Therefore, the antiradical properties of any substance may play an important role in the reduction of inflammatory nociception [32]. In our study, compounds **13b**, but not **10b**, at the medium and high doses decreased MPO level compared to the control group. It may result from the fact that substance **13b** exerts antiradical activity, as revealed in our earlier in vitro studies [6,10]. Meanwhile, compound **10b** does not lower ROS level and even increases the RNS amount, presumably due to the nitric substituent in the phenyl ring of the arylpiperazine pharmacophore. Nevertheless, we have not proven the exact impact of the substituent in arylpiperazine or arylpiperidine on the level of ROS or RNS yet.

Prostaglandins, as pain sensitizing agents, are the primary target of analgesics, which block their synthesis by inhibiting COX activity (NSAIDs). Since PGs also play a key role in several physiological processes, including gastroprotection, non-selective inhibition of COX-1 and COX-2 derived prostanoids, due to long-term use of non-specific NSAIDs, may lead to serious side effects such as gastrointestinal irritation, bleeding, and ulceration. It should be pointed out that not only the mechanism of action, but also the structural properties of NSAIDs contribute to their gastrotoxicity. The presence of free carboxylic moiety in the structure of most NSAIDs induces mucosal damage by local irritation. In the acidic stomach environment, these drugs remain in lipophilic, non-ionized form, but they dissociate while entering the cells, and the effect of ionic trap occurs. This, in turn, induces gastric mucosal lesions. Moreover, diminished hydrophobicity of gastric mucosa makes it more susceptible to the harmful effects of hydrochloric acid [33,34]. In this study, the macroscopic stomach analysis revealed that both tested compounds showed a superior profile in regard to gastric injuries as compared to indomethacin. These findings corroborated with results of the histological assessment of the gastric mucosa and are consistent with findings reported by other authors who have shown that 1,3,4 oxadiazole-2-thione ring incorporated into a given structure results in lower gastrotoxicity [7,8]. Noteworthy, novel compounds exerted an antinociceptive effect comparable with indomethacin with the advantage of having no ulcerogenic activity.

**Figure 8:**
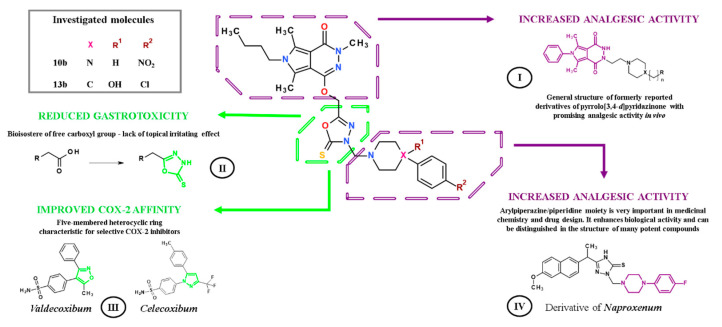
The design concept of the novel pyrrolo[3,4-*d*]pyridazinone derivatives.

When considering the molecule of discussed derivatives, we can focus on some characteristic structural elements, which with a high degree of probability are responsible for their biological activity and low gastrotoxicity (Figure 8). First of all, different compounds based on pyrrolo[3,4-*d*]pyridazinone core have already been extensively investigated in vitro and in vivo, as well (Figure 8, I). Some of them exerted significant antinociceptive activity, but their exact mechanism of action has not been solved yet [4,5,35]. This encouraged us to perform further structural modifications of this promising biheterocyclic scaffold [6]. The introduction of the five-membered 1,3,4-oxadiazole-2-thione ring was expected to enhance the COX-2 affinity and reduce gastrointestinal side effects of the final compounds (Figure 8, II, III) [7,8,9,36,37]. This is one of the most important pharmacophores in current medicinal chemistry, serves as bioisostere for carboxyl group, and is present in many bioactive molecules. According to the literature, replacement of free carboxyl, which is characteristic for most of the NSAIDs, with 1,3,4-oxadiazole-2-thione, results in better COX-2 affinity and substantially reduced gastrotoxicity of designed compounds (Figure 8, II) [7,8,9,36,37]. Moreover, when compared with free carboxylic moiety, the oxadiazole nucleus enhances the interaction with cyclooxygenase receptor by the formation of numerous hydrogen bonds [38]. Such modifications were successfully performed on the skeleton of ibuprofen [7], diclofenac [9], or naproxen [37]. Moreover, this moiety precisely refers to the characteristic five-membered heterocyclic ring present in the structure of COX-2 selective inhibitors (Figure 8, III) [33,36]. Finally, the arylpiperazine or arylpiperidine pharmacophore has been introduced to the structure of titled compounds via Mannich reaction (Figure 8, IV). The presence of this bioactive group is identified with enhanced pharmacological activity of designed pyrrolo[3,4-*d*]pyridazinone derivatives. Arylpiperazine or arylpiperidine pharmacophore, which are present in the structure of investigated molecules **10b** and **13b**, can also be distinguished in many drugs and new drug candidates (Figure 8, IV) [4,5,6,7,8,9,10,35,36,37,39,40]. According to literature reports, the introduction of the electron-withdrawing substituent in position 4 of the aryl ring in that moiety often results in enhanced pharmacological activity of the final compounds. By analogy to this premise, the title compounds **10b** and **13b** are bearing nitro or chlorine residue [7,8,9].

## 4. Materials and Methods

### 4.1. Drugs and Chemicals

The studied compounds, novel pyrrolo[3,4-*d*]pyridazinone derivatives, named **10b** and **13b**, were synthesized from commercially available reagents and solvents according to the previously described method [6]. The structures and purity of **10b** and **13b** were confirmed by different spectroscopic techniques: 1H NMR, 13C NMR, MS, FTIR, elemental analysis, and on the basis of their physicochemical properties.

Drugs and chemicals used in the experiment were as follows: Indomethacin (Sigma-Aldrich, Steinheim, Germany); morphine hydrochloride (Fagron, Kraków, Poland); carboxymethylcellulose (CMC) (PolAura, Olsztyn, Poland) dissolved to 0.5% solution in normal saline (Polpharma, Starogard Gdański, Poland); pentobarbital sodium + pentobarbital 133.3 mg/mL + 26.7 mg/mL, sol. (Morbital, Biowet, Puławy, Poland); medetomidine hydrochloride 1 mg/mL, sol. (Domitor, OrionPharma, Warszawa, Poland); formalin 37% sol. (Chempur, Piekary Śląskie, Poland) dissolved to 1.5% solution in normal saline (as above). Other chemicals used were included in the commercially available kits.

### 4.2. Animals

Male Wistar rats (233.08 ± 18.83 g) and Swiss albino mice (45.35 ± 3.06 g) were purchased from the Animal Research Center at Wroclaw Medical University (Wrocław, Poland) and throughout the acclimatization and study periods kept in pairs in transparent polypropylene cages with water ad libitum. All animals were kept in standard laboratory conditions with 12 h light/dark cycle, a temperature of 21–24 °C, a humidity of 55–60%, and free access to standard rodent chow (Agropol, Motycz, Poland), except for a single procedure of deprivation. Animals were accustomed to the laboratory condition for 7 days before the experiment’s beginning.

### 4.3. Ethics Statement

The animal care and all experimental procedures were in accordance with the applicable international, national, and institutional guidelines, including the Act of 15 January 2015 on the protection of animals used for scientific and educational purposes (Journal of Laws of 2015, item 266) and EU directive 2010/63/EU. All experiments were conducted with the permission (Resolution No. 101/2018 of 12.12.2018) of the Local Ethics Committee for Animal Experiments in Wrocław at Hirszfeld Institute of Immunology and Experimental Therapy of Polish Academy of Sciences (53-114 Wrocław, Weigla 12, Poland).

### 4.4. Drug Administration

Animals were food-deprived for 12 h before appropriate substance administration. Studied substances were suspended in 0.5% CMC solution and given in a single dose intragastrically by the gastric tube (FST, Foster City, CA, USA) in a volume of 4 mL/kg. The solution of 0.5% CMC in control groups was given by the same route and in equivalent volume. Doses of studied substances, morphine and indomethacin, were chosen based on the earlier works [5,35,41,42]. Animals were sacrificed by intramuscular injection of medetomidine (0.5 mg/kg) followed by intraperitoneal injection of pentobarbital (200 mg/kg).

### 4.5. Tail-Flick Test

Rats were randomly divided into 8 experimental groups of 12 animals, each organized as follows: A control group receiving 0.5% carboxymethylcellulose solution; groups receiving compound **10b** or **13b** (5, 10, 20 mg/kg), and the group receiving morphine (10 mg/kg) as a reference drug. One hour after administration of the appropriate experimental substance, the tail-flick test was performed using the tail-flick apparatus (TF-01, Porfex, Białystok, Poland) according to D’amour and Smith with slight alterations [43]. Each rat was gently restrained by hand, its tail was placed in a slot of variable width supplied with a groove that guarantees a precise placement and enables free movement, and the beam (thermal stimulus) was directed onto the tail about 4–6 cm from the tip. The antinociceptive effect of studied substances was measured as the reaction time from thermal stimulus application to tail withdrawal (latency time). A maximum latency time (cut-off time) of 10 s was set in order to avoid tissue harm. The allocation of animals was concealed, and the result was blindly assessed during the tail-flick test.

### 4.6. Formalin Test

Mice were randomly divided into 9 experimental groups of 12 animals each as follows: Control group receiving 0.5% carboxymethylcellulose solution; experimental groups receiving compound **10b** or **13b** (5, 10, 20 mg/kg), and groups receiving references drug—indomethacin (10 mg/kg) or morphine (10 mg/kg). One hour after administration of the appropriate experimental substance, the formalin test was carried out as described by Hunskaar and Hole [15]. Mice were injected subcutaneously using a microsyringe with a 26-gauge needle with 0.02 mL of 1.5% formalin solution to the dorsal surface of the right hind paw. The pain reaction was measured as the duration of licking, shaking, and biting (in seconds) of the injected paw during the neurogenic (early; 0–5 min) and inflammatory (late; 25–30 min) phase of the nociceptive reaction. The time of pain response was recorded with a handheld stopwatch by 3 independent observers with animal allocation concealment and blind outcome assessment. Blood samples from the tail vein and stomachs were collected, and then the mice were sacrificed.

### 4.7. Evaluation of PGE_2_ and MPO Levels

The concentrations of PGE_2_ and MPO were measured in mice serum with enzyme-linked immunosorbent assay (ELISA) kits: Mice PGE_2_ Elisa Kit, Mice MPO Elisa Kit (Cloud-Clone Corp., Katy, TX, USA) following the manufacturer’s instructions. All concentrations were expressed as pg/mL.

### 4.8. Histopathological Assessment of Gastric Mucosa

The mucosal damage was measured using the macro- and microscopic examination. The stomach from each mouse was removed, opened along the greater curvature, and cleaned gently by dipping in normal saline. The severity of macroscopically visible changes in the mucous membrane was assessed using the semiquantitative 0–3 scale based on the previously described criteria taking into account the presence or absence of gastric erosions, ulcers, and their extent, described in detail in the legend of Table 1 [44]. Then, stomach specimens demonstrating macroscopic injury of the greater extent collected from each mouse pretreated with studied compound or reference drug, or equivalent stomach specimens from control mice, were selected for the histological assessment. Selected specimens were fixed in 4% buffered formalin, embedded in paraffin, and cut into 4 µm-thick slices, which were mounted on the glass slides and stained by the routine hematoxylin-eosin (H&E) method. Next, the microscopic analysis was performed using an Olympus BX53 light microscope combined with an Olympus UC-90 camera (Olympus, Germany). Histopathological changes of all stomach specimens were examined in a blinded way by the experienced pathologist using a 0–3 point scale.

### 4.9. Statistical Analysis

All experimental data are presented as mean values ± standard error of the mean (SEM). Statistical differences between studied parameters were analyzed using one-way analysis of variance (ANOVA) and multiple comparisons with Tukey’s post hoc test. Comparison of the activity of the tested compounds was performed with multi-criteria decision analysis (MCDA) using the weighted sum model (WSM). The weights were selected based on the meaning of each bioassay. The weights were set at 0.2 for the tail-flick test, formalin test (phase 1, phase 2), and the assessment of PGE_2_ and MPO levels. All statistical analyses were performed with Statistica v. 13.1 (StatSoft, Kraków, Poland) with statistical significance set at *p*-value < 0.05.

## 5. Conclusions

Based on the results of the present study, the novel pyrrolo[3,4-*d*]pyridazinone derivatives **10b** and **13b** exert dose-dependent antinociceptive activity and a lack of gastrotoxicity. Moreover, studied molecules counteract inflammatory nociception. The findings from multi-criteria decision analysis imply that both studied compounds act more efficiently than indomethacin, but not morphine. Compound **13b** at the high dose (20 mg/kg) reveals the greatest antinociceptive effect among all studied doses of two novel compounds. Thus, compound **13b** might be considered as the most promising analgesic agent in conditions of pathological pain, including inflammatory pain. The beneficial effect observed in the case of compound **13b** could be attributed to its COX-2 selectivity and ability to reduce nociceptor sensitization via PGE_2_ and MPO levels decrease. The results obtained in this study prove that new pyrrolo[3,4-*d*]pyridazinone derivatives constitute a promising scaffold for further development into potent and safe analgesic agents. Further work needs to be carried out to thoroughly study and confirm the mechanism involved in the antinociceptive effect of examined compounds.

Considering that compounds **10b** and **13b** exert an antinociceptive effect in the late phase of the formalin test, it might be assumed that they also possess anti-inflammatory action. Further research focusing on the anti-inflammatory activity of novel pyrrolo[3,4-*d*]pyridazinone derivatives and their potential mechanisms, as well as assessing safety profile in regard to gastrotoxicity in long-term administration, is already underway.

## Figures and Tables

**Table 1:** The impact of compounds **10b** and **13b** on gastric mucosa. Morphine and indomethacin were used as reference drugs. Experimental groups (*n* = 12): C—control group; M—group receiving 10 mg/kg of morphine; I—group receiving 10 mg/kg of indomethacin; **10b**-5, **10b**-10, **10b**-20—groups receiving, respectively, 5, 10, or 20 mg/kg of investigated compound **10b**; **13b**-5, **13b**-10, **13b**-20—groups receiving, respectively, 5, 10, or 20 mg/kg of investigated compound **13b**. Scoring scale of macroscopic evaluation of gastric mucosa: (0) = no damage; (1) = 1–4 small petechiae; (2) = 5 or more petechiae or hemorrhagic streaks up to 4 mm; (3) = erosions longer than 5 mm or confluent hemorrhages. Scoring scale of microscopic evaluation of gastric mucosa: (0) = no damage; (1) = mild changes; (2) = moderate changes; (3) = severe changes. Data are presented as mean values ± SEM. Differences ^^^ *p* < 0.001 vs. the control group; *** *p* < 0.001 vs. the indomethacin group were deemed statistically significant.

Group	Macroscopic Lesions (0–3 Points)	Microscopic Lesions, H&E Staining (0–3 Points)
control	0	0
M	0	0.43 ± 0.21
I	2.00 ± 0.31 ^^^	2.43 ± 0.30 ^^^
**10b**-5	0	0
**10b**-10	0	0.29 ± 0.18
**10b**-20	0.57 ± 0.20 ***	0.71 ± 0.29 ***
**13b**-5	0	0
**13b**-10	0	0
**13b**-20	0.29 ± 0.18 ***	0.57 ± 0.20 ***
